# VISTA is a potential target for immunotherapy in B-cell acute lymphoblastic leukemia in children

**DOI:** 10.1038/s41598-025-08164-2

**Published:** 2025-07-03

**Authors:** Nourhan K Mohamed, Mohamed A El-Mokhtar, Asmaa M Zahran, Gamal Fadl Mahmoud Gad, Reham Ali Ibrahem

**Affiliations:** 1 Department of Microbiology and Immunology, Faculty of Pharmacy, Minia University, Minia, 61519 Egypt; 2 Department of Microbiology and Immunology, Faculty of Pharmacy, Sphinx University, New Assiut City, 71515 Assiut Egypt; 3 Gilbert & Rose-Marie Chagoury School of Medicine, Lebanese American University, P.O. Box 36, Byblos, Lebanon; 4 Department of Clinical Pathology, South Egypt Cancer Institute, Assiut, University, Assiut, 71515 Egypt

**Keywords:** B-cell acute lymphoblastic leukemia, VISTA, CD244, CD48, FOXD3, PVRL2, Biochemistry, Biological techniques, Cancer, Immunology

## Abstract

Immune checkpoints expressed by immune cells are critical mediators of immune suppression in cancer. Recently, the role of VISTA, an immune checkpoint, in suppressing T cells has been highlighted in several studies. However, its involvement in B-cell acute lymphoblastic leukemia (B-ALL) remains underexplored. In this study, we analyzed the expression of VISTA, the immune-regulatory marker CD244, and its corresponding ligand CD48. Additionally, we examined the expression of the transcription factors FOXD3 and PVRL2 in pediatric patients with B-ALL. Peripheral blood samples from pediatric patients with naïve B-ALL were analyzed using flow cytometry. Additionally, real-time PCR was used to evaluate the downstream regulatory gene FOXD3 expression and the immune regulator PVRL2. VISTA was overexpressed on blasts in B-ALL patients. Also, the frequency of CD3^+^CD8^+^VISTA^+^ T cytotoxic cells was significantly higher in patients compared to controls, while CD3^+^CD4^+^VISTA^+^ T helper cells were reduced. CD19^+^VISTA^+^ cells were more abundant in the complete remission group compared to the non-complete remission group. FOXD3, a key regulator of VISTA, was significantly downregulated in B-ALL, consistent with VISTA overexpression. The CD244/CD48 interaction, which can promote anti-tumoral immune responses, showed a reduction in CD3^+^CD4^+^CD48^+^ and CD19^+^CD48^+^ cells, while CD3^+^CD8^+^CD48^+^ cells were increased. VISTA overexpression and FOXD3 downregulation in B-ALL, alongside altered CD48, and PVRL2 expression, highlight mechanisms of immune evasion. These findings position VISTA as a promising biomarker and target for B-ALL immunotherapy.

**Supplementary Information:**

The online version contains supplementary material available at 10.1038/s41598-025-08164-2.

## Introduction

Acute lymphoblastic leukemia (ALL) is a hematological malignancy of B or T lymphocytes characterized by increased activity and clonal proliferative capacity, resulting in the accumulation of neoplastic lymphocytes in the blood, bone marrow, and lymphoid tissues. This leads to lymphocytosis, marrow infiltration, lymphadenopathy, and splenomegaly. ALL is the most common cancer in the pediatric population, with B-ALL being the most aggressive and prevalent subtype, accounting for approximately 80% of cases^1^. Even though the cure rate is up to 90% ^2^, chemoresistance^3^treatment failure and relapse rates ranging from 9.5 to 43% occur^4^. Furthermore, most of the adverse events encountered which are severe or higher, are a major cause of hospital readmissions^5^ and are a significant cause of treatment-related mortality^6^.

Cancer employs several immune evasion mechanisms to suppress the host’s immune responses. One important mechanism that has been extensively investigated is the upregulation of immune checkpoints (ICs). These are inhibitory receptors that negatively regulate immune responses^7^. Of these molecules; PD-1, CTLA-4, TIM-3, TIGIT, VISTA and CD244 ^8^. They become upregulated in chronic viral infections and cancer; they are markers of exhausted T cells. Their role is increasingly emphasized and is being targeted for therapeutic purposes. T cytotoxic, T helper, and T regulatory cells (Tregs) greatly impact leukemia cell survivability, cancer cell proliferation, immune suppression, and patient prognosis. A better understanding of the IC expression, binding ligands expression and downstream regulatory proteins expression in B-ALL can aid in drug design of molecularly-targeted drugs, and reduce adverse events and chemoresistance encountered with non-targeted therapeutics.

VISTA is a transmembrane protein; it is short for V- Domain Ig suppressor of T cell activation, also known as (B7-H5). VISTA acts as a ligand expressed on antigen-presenting cells and as a receptor expressed on T cells, its role in immune regulation was recognized, and its possible utilization in immunotherapy is proposed. It has an important role in immune regulation, it suppresses the anti-tumoral activity and proliferative capacity of T cells^9–12^. It inhibits the production of IL-2 by CD4^+^ and INF-γ by CD8^+ 11^. Accumulating evidence described the role of VISTA as an IC and its expression was high in melanoma^13^non-small cell lung cancer^14^acute myeloid leukemia^15–17^colorectal cancer, pancreatic adenocarcinoma and gliomas^18^. Preclinical models showed an anti-tumoral activity when anti-VISTA monoclonal antibody was tested; it restored T cell activity and enhanced NK-mediated monocyte activation^19^. Clinical trials testing anti-VISTA including, NCT04475523 and NCT05864144 showed improvements in therapeutic outcomes^20^.

FOXD3 (forkhead box D3) is a transcription factor that is important in regulating differentiation and embryonic cell self-renewal^21^. It was previously indicated that FOXD3 suppresses the expression of VISTA in melanoma cells via the BRAF/MEK/ERK signaling pathway^22^. Its expression was altered in CLL^23,24^gliomas^25^and melanoma^22^. FOXD3 contributed to leukemogenesis, cancer cell proliferation and poor patient’s prognosis^25–27^.

Cluster of Differentiation 244 (CD244) also known as Natural Killer Cell Receptor 2B4 (NKR2B4) is an immunoregulatory transmembrane receptor that belongs to the Signaling Lymphocyte Activation Molecule (SLAM) family. Normally, its expression was frequently reported on natural killer (NK) cells, γδ T cells, basophils, monocytes, a subset of CD8^+^ αβ T cells which mediate non-MHC-restricted cytotoxicity, dendritic cells (DC), and myeloid-derived suppressor cells (MDSC)^28,29^. CD244 has been recognized as an IC; it was expressed by exhausted T cells in AML and chronic lymphocytic leukemia (CLL)^30^. Its interaction with its corresponding ligand is important in stimulating NK-mediated cytotoxicity.

Cluster of Differentiation 48 (CD48) is also called B-lymphocyte activation marker (BLAST-1). It is one of the signaling lymphocytic activation molecule family SLAMF2. It is the corresponding binding ligand for CD244. Their interaction will stimulate NK cells^31^. Usually, CD48 is present in all peripheral blood lymphocytes (PBL); T lymphocytes, B lymphocytes as well as NK cells^32^. It is also found on the surface of activated T cells, mast cells, monocytes, and granulocytes. It is the ligand for the aforementioned CD244; upon interaction, CD244 stimulates NK cells^31^.

Poliovirus receptor-related 2 (PVRL2), also called CD112, is an immune regulator that binds to T cell immunoreceptors with Ig and ITIM domains (TIGIT). It is an IC that is frequently upregulated by tumors. TIGIT was found to be highly expressed by the circulating non-Treg T cells from adult B-ALL^33^ PVRL2 was highly expressed by AML cell lines and patient samples. When the TIGIT-PVR/PVRL2 axis was blocked, improved therapeutic outcomes were observed^34^.

Despite the critical role of VISTA as a novel immune checkpoint in mediating immune suppression and its potential as a therapeutic target in B-ALL, its role in pediatric patients remains underexplored. This study aimed to investigate the expression of VISTA in pediatric B-ALL patients and its contribution to immune evasion. Additionally, the study examines the expression and interactions of key immune regulatory markers, including CD244, CD48, FOXD3, and PVRL2, to provide a comprehensive understanding of the immune landscape in B-ALL.

## Materials and methods

### Study participants

#### Ethical approval

The experimental protocol was approved by the Minia University Faculty of Pharmacy Ethics Committee under approval number MPEC (230505), and all methods were performed in accordance with the relevant guidelines and regulations.

#### Patients and study setting

The patients were admitted to the South Egypt Cancer Institute between November 2021 and March 2023. Peripheral blood (PB) samples were collected upon admission. The study included 37 newly diagnosed, untreated pediatric patients with B precursor-ALL (B-ALL) (median age: 5 years; range: 1–15 years). Patients with central nervous system involvement or suffering from any malignant diseases other than B-ALL were excluded. Diagnosis was established based on clinical presentation, complete blood picture, differential white blood cell count, bone marrow examination, immunophenotyping, and cytogenetics. Immunophenotyping of leukemic blasts from bone marrow aspirate (MBA) was done using known surface markers, including CD10, CD19, CD3, CD7, CD8, CD4, CD34, CD38, CD45, CD79a, HLA-DR, and Cyto IgM by flow cytometry.

Patients were divided into two groups according to their prognosis: complete remission (CR) and non-complete remission (NCR). Patients were tested for measurable/minimal residual disease (MRD) using CD10, CD19, CD38 and CD45.

Moreover, PB samples were also collected from a control group consisting of 25 healthy, age- and sex-matched children, who had no known hematologic or malignant conditions, in order to provide a baseline for comparison with the patient group.

### Sample collection and processing

Peripheral blood mononuclear cells (PMCs) were isolated from freshly collected peripheral blood samples using Ficol Hypaque medium (Biowest, UK) by density gradient centrifugation. PMCs were then washed and used for flow cytometry and Real-time PCR (RT-PCR) analyses as described below.

### Flow cytometry

The expression of immune checkpoints VISTA, CD244, and CD48 (the ligand for CD244) was analyzed on CD3 + CD4 + T cells, CD3 + CD8 + T cells, and CD19 + B cells using flow cytometry. Cell acquisition and flow cytometric evaluation were carried out by FACS Canto™ flow cytometer with FACS Diva software (BD Biosciences, San Jose, California, USA) at the Flow cytometry Unit, Clinical Pathology Department, South Egypt Cancer Institute. Data was analyzed using FlowJo™ software V10.9 (BD Biosciences, Ashland, OR, USA).

Peripheral mononuclear cells (PMCs) were stained using the following monoclonal antibodies: CD19 APC-H7 (Elabscience, USA), CD3 V450 (Elabscience, USA), CD4 PerCP/Cyanine5.5 (Elabscience, USA), CD8a (FITC-CD8a) (Elabscience, USA), VISTA conjugated Phycoerythrin - Cyanine7 (PE-Cy7-VISTA) (Invitrogen, USA), CD48 conjugated allophycocyanin (APC-CD48) (Elabscience, USA), CD244 conjugated Phycoerythrin (PE-CD244) (Invitrogen, USA).

About 50 µl of blood sample were incubated with 5 µl of CD19 APCH7, CD3 V450, CD4 PerCP/Cyanine5.5, FITC-CD8a, PE-Cy7-VISTA, APC-CD48, PE-CD244 monoclonal antibodies for 15 min at 4 °C in dark. Lysing solution was then added to the mixture followed by centrifugation at 1500 rpm for 1 min, and the supernatant was discarded. The mixture was then washed by PBS followed by centrifugation at 1500 rpm for 1 min, and the supernatant was discarded. The cells were resuspended in PBS following washing. Isotype-matched controls were included for each sample. All experiments were conducted on freshly collected samples. Representative gating strategy for the main lymphocyte subsets and VISTA expression is shown in supplementary Fig. 1.

### RT-PCR analysis

The total RNA was extracted using the Trizol-chloroform method using the ABT Total RNA Mini Extraction Kit (Applied Biotechnology, Egypt). cDNA was synthesized according to the manufacturer’s instructions using ABT 2X RT mix (Applied Biotechnology, Egypt). The expression of the transcription factor FOXD3 (regulator of VISTA), PVRL2 (ligand for TIGIT) and GAPDH (reference gene) were analyzed by RT-PCR using Syber green master mix (HERA^Plus^ Syber^®^ Green qPCR kit, Willofort, UK). Primers for each gene were obtained from Macrogen (South Korea). Sequences for forward and reverse primers are shown in Table 1. The reaction was carried out in the Reproductive Science Research Center, Assiut University using Gentier-48E thermocycler instrument. RT-PCR program was initiated with denaturation at 95 ^o^C for 3 min followed by 40 cycles of amplification at 95 ^o^C for the 30 s, 60 ^o^C for 30 s, and final extension at 72 ^o^C for 1 min. Measurements were performed in duplicates, and fold change gene expression was calculated by relative quantification using 2^−ΔΔCT^ (Livak method).

**Table 1:** Primer sequences used for RT-PCR Analysis.

Gene	Role/implication	Forward	Reverse	References
PVRL 2	The gene coding for a ligand for TIGIT	5’-GAGGACGAGGGCAACTACAC-3’	5’-AGGGATGAGAGCCAGGAGAT-3’	^34^
FOXD3	The gene coding for transcription factor regulating VISTA	5’-CCCTACTACAGGGAGAAGTTCCC-3’	5’-CGGGTCCAGGGTCCAGTA-3’	^35^
GAPDH	Reference gene	5’-GGT CAC CAG GGC TGC TTTTA-3’	5’-TTC CCG TTCTCA GCC TTGAC-3’	^34^

### Statistical analysis

Statistical analyses were performed using the GraphPad Prism 8.4.3 software (GraphPad Software, La Jolla, CA, USA). Medians, means, and standard deviations were calculated. The Mann-Whitney U test was used to evaluate differences in the percentages of cell subsets. The A Student’s t-test was performed to assess differences in mRNA levels between patient and control specimens for each investigated marker. Pearson’s correlation coefficient test was used to analyze the linear association between cell subsets. A two-tailed p-value of less than 0.05 was considered significant.

## Results

### Patient characteristics

The study cohort included 37 newly diagnosed, untreated pediatric patients with B-cell acute lymphoblastic leukemia (B-ALL). The median age of the patients was 5 years (range: 1–15 years), with a male-to-female ratio of 1.64:1 (62.2% male and 37.8% female). The median percentage of blasts in the bone marrow was 88.35% (range: 56–97%). Platelet counts varied widely, with a median of 40 × 10⁹/L (range: 2–192 × 10⁹/L). Patients were categorized into two prognostic groups based on treatment response: 22 patients (59.5%) achieved complete remission (CR), while 15 patients (40.5%) were classified as non-complete remission (NCR). Table 2 summarizes these demographic and clinical characteristics.

**Table 2:** Characteristics of pediatric B-ALL patients.

Characteristic	Counts
Patients - n	37
Sex- n (%)	
Male	23 (62.2)
Female	14 (37.8)
Age (years)	
Median	5
Range	1–15
Blasts (%)	
Median	88.35
Range	56–97
Average platelet count(10^9^/L)	
Median	40
Range	2-192
Prognosis (n)	
CR	22
NCR	15

CR: Complete remission, NCR: Non-complete remission. Values for blasts and platelet counts are presented as medians with ranges.

### Altered distribution of lymphocyte subsets

**Fig. 1:**
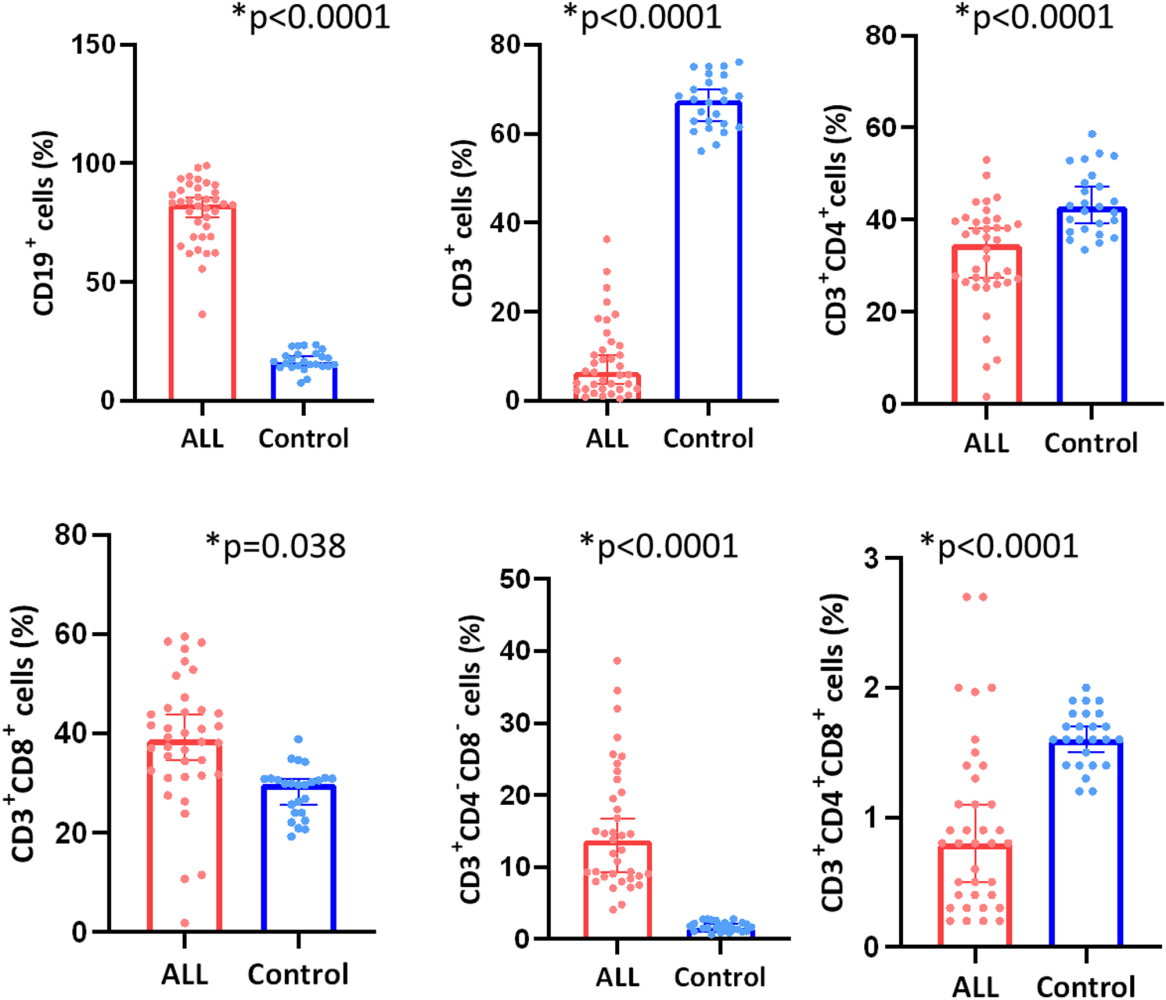
Comparative analysis of the main lymphocyte subsets in B-ALL patients and healthy controls. The plots show the mean frequencies of the indicated cell subsets in B-All patients (red) compared to controls (blue) ± standard deviation. Error bars represent the standard deviation. Comparisons were carried out using the Mann-Whitney U test. Statistical significance is denoted with *p < 0.05.

First, the distribution of lymphocyte subsets in B-ALL patients was analyzed using flow cytometry and compared to healthy controls. A significant increase in the percentage of the CD19^+^ B-cell population was observed in B-ALL patients compared to controls (82.9% vs. 16.6%, *p* < 0.0001). In contrast, the percentage of CD3^+^ T cells was markedly reduced in B-ALL patients (6.4% vs. 67.5%, *p* < 0.0001), as shown in Fig. 1.

Within the CD3^+^ population, the proportion of CD3^+^CD4^+^ T helper cells was significantly decreased in B-ALL patients compared to controls (34.65% vs. 42.9%, *p* < 0.0001), while the percentage of CD3^+^CD8^+^ cytotoxic T cells was significantly increased (38.75% vs. 29%, *p* = 0.038). Additionally, the double-negative CD3 subset (CD3^+^CD4^−^CD8^−^ T cells), known to play a role in cancer progression, was significantly elevated in B-ALL patients compared to controls (15.41% vs. 1.77%, *p* < 0.0001). Conversely, the double-positive CD3 subset (CD3^+^CD4^+^CD8^+^ T cells) was significantly reduced in B-ALL patients compared to controls (0.94% vs. 1.61%, *p* < 0.0001), highlighting a notable alteration in T-cell distribution in B-ALL.

### Expression of VISTA in B-ALL

**Fig. 2:**
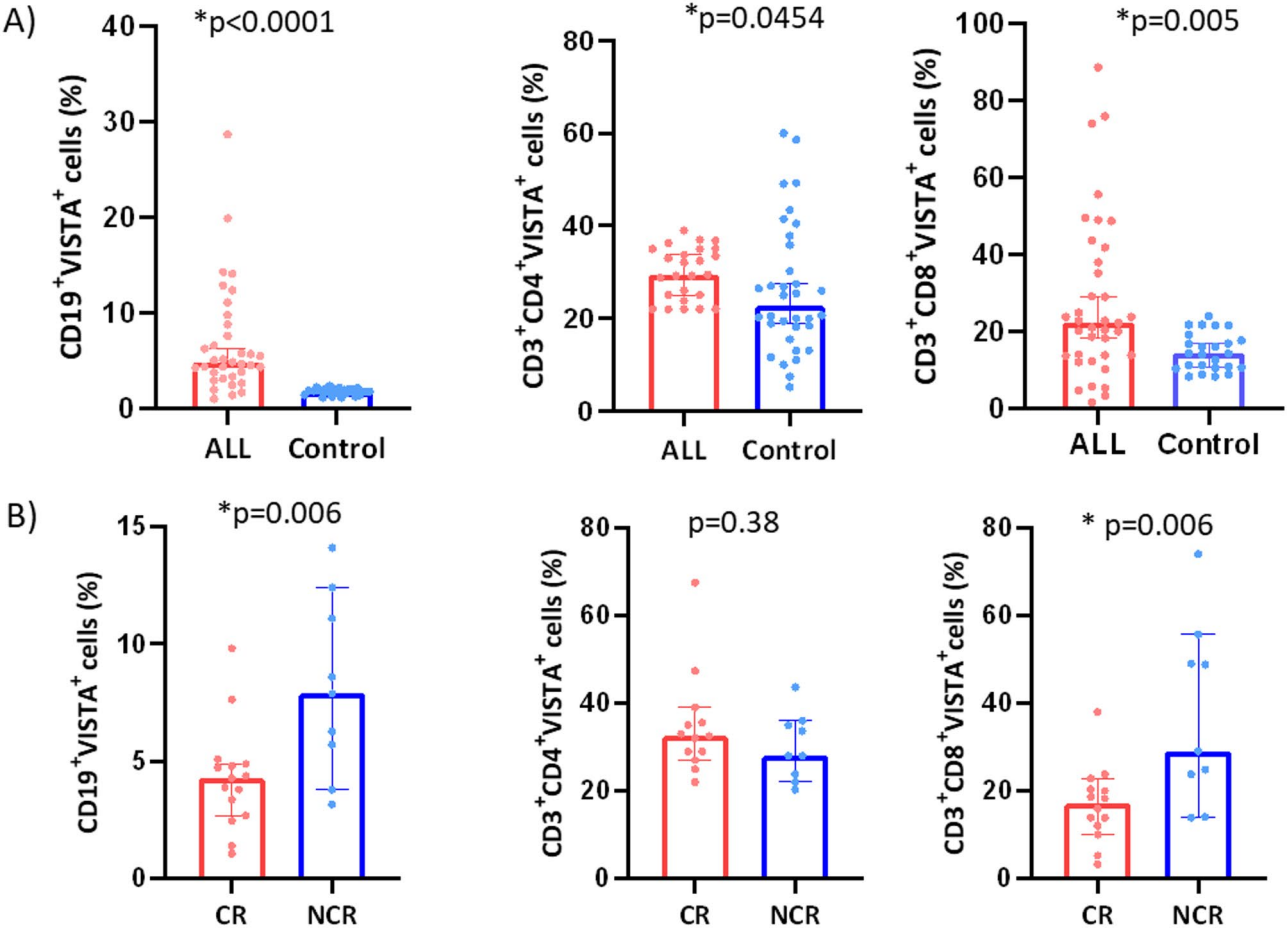
VISTA expression on lymphocyte subsets and in remission groups in B-ALL patients. (**A**) VISTA expression on indicated lymphocyte subsets in B-ALL patients (red) compared to healthy controls (blue, *n* = 9). (**B**) Comparison of VISTA expression on lymphocytes between complete remission (CR, *n* = 15) and non-complete remission (NCR, *n* = 9) groups of B-ALL patients. The bars represent the mean ± standard deviation (SD). Statistical analysis was carried out using the Mann-Whitney U test. Statistical significance is denoted with *p < 0.05.

To investigate the role of VISTA in B-ALL, we analyzed its expression across lymphocyte subpopulations, including CD19^+^ B cells, CD3^+^CD4^+^ T helper cells, and CD3^+^CD8^+^ cytotoxic T cells. Our findings revealed that CD19^+^VISTA^+^ cells were significantly more abundant in B-ALL patients compared to healthy controls (4.88% vs. 1.86%, *p* < 0.0001). In T lymphocytes, VISTA expression was also significantly upregulated, with higher levels observed on CD4^+^ T cells (29.83% vs. 24.59%, *p* = 0.045) and CD8^+^ T cells (22.55% vs. 14.3%, *p* = 0.005) in B-ALL patients compared to controls (Fig. 2A). Malignant cells employ multiple immune evasion mechanisms; one of them is to express ICs like VISTA to attenuate T-cell activity.

### VISTA expression in complete remission groups versus noncomplete remission group

Furthermore, patients were divided into complete remission (CR) and non-complete remission (NCR) subgroups. The percentage of blasts expressing VISTA^+^ in the CR group was significantly lower than in patients with NCR (4.28% vs. 8.69%, *n* = 21, *p* = 0.025), implying a possible immunoinhibitory effect. Moreover, the CD8^+^VISTA^+^ cells were reported to be significantly lower in the CR group vs. NCR group (16.1% vs. 24.9%, *p* = 0.034) as shown in Fig. 2B.

To evaluate the risk of relapse, the B-ALL patients were tested for MRD using monoclonal antibodies against CD10, CD19, CD38, and CD45. Cases that reported MRD levels below 0.01% in their bone marrow samples were considered negative. Accordingly, patients were divided into positive MRD and negative MRD. However, no significant difference was found between the two groups regarding VISTA, CD48, or CD244 surface expression.

### CD244 expression by lymphocytes

CD244 is a regulatory immune receptor expressed by various immune cells and associated with cell exhaustion. When CD244 expression was analyzed on B and T cells, no significant changes were reported between B-ALL patients and controls.

### Differential CD48 expression

**Fig. 3:**
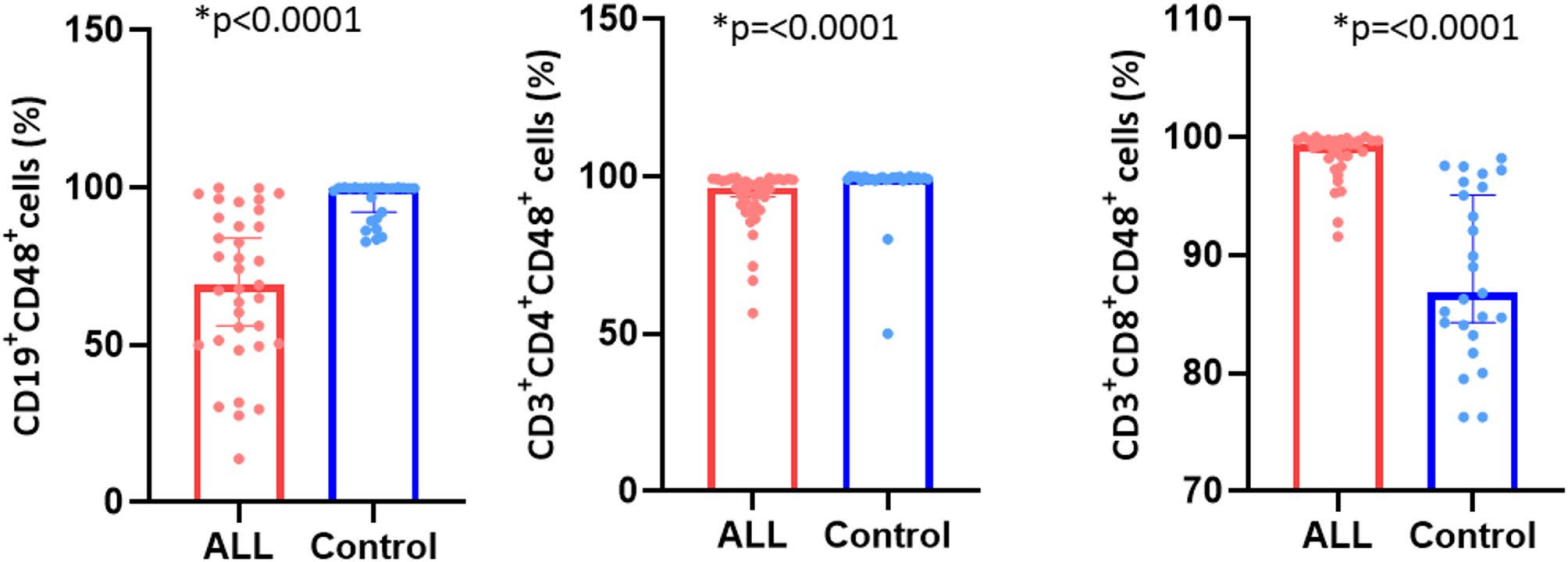
CD48 expression on lymphocyte subsets in B-ALL patients and healthy controls. Bar plots with individual data points showing CD48 expression on CD19 + B cells, CD3 + CD4 + T helper cells, and CD3 + CD8 + cytotoxic T cells in B-ALL patients (red) compared to healthy controls (blue). The bars represent the mean ± standard deviation (SD). Statistical analysis was performed using the Mann-Whitney U test. Statistical significance is denoted with *p < 0.05.

Interestingly, CD48 (the ligand for the CD244 receptor) was significantly downregulated on B-cells in B-ALL patients compared to controls (68.7 ± 21.7% vs. 95.6 ± 6.3%, *p* < 0.0001), as shown in Fig. 3. Similarly, the percentage of CD3 + CD4 + CD48 + cells was significantly lower in B-ALL patients than in controls (92.7 ± 9.7% vs. 96.7 ± 10.3%, *p* < 0.0001). Conversely, CD3 + CD8 + CD48 + cells were significantly increased in B-ALL patients compared to controls (98.5 ± 2% vs. 88.5 ± 7.1%, *p* < 0.0001). Reduced CD48 expression by tumor cells may impair CD244-CD48 interactions, rendering them less recognizable by natural killer (NK) cells and cytotoxic T lymphocytes (CTLs). This mechanism likely facilitates immune evasion by tumor cells.

### Dysregulated expression of FOXD3 transcription tactor and PVRL2 immune regulators in B-ALL

**Fig. 4:**
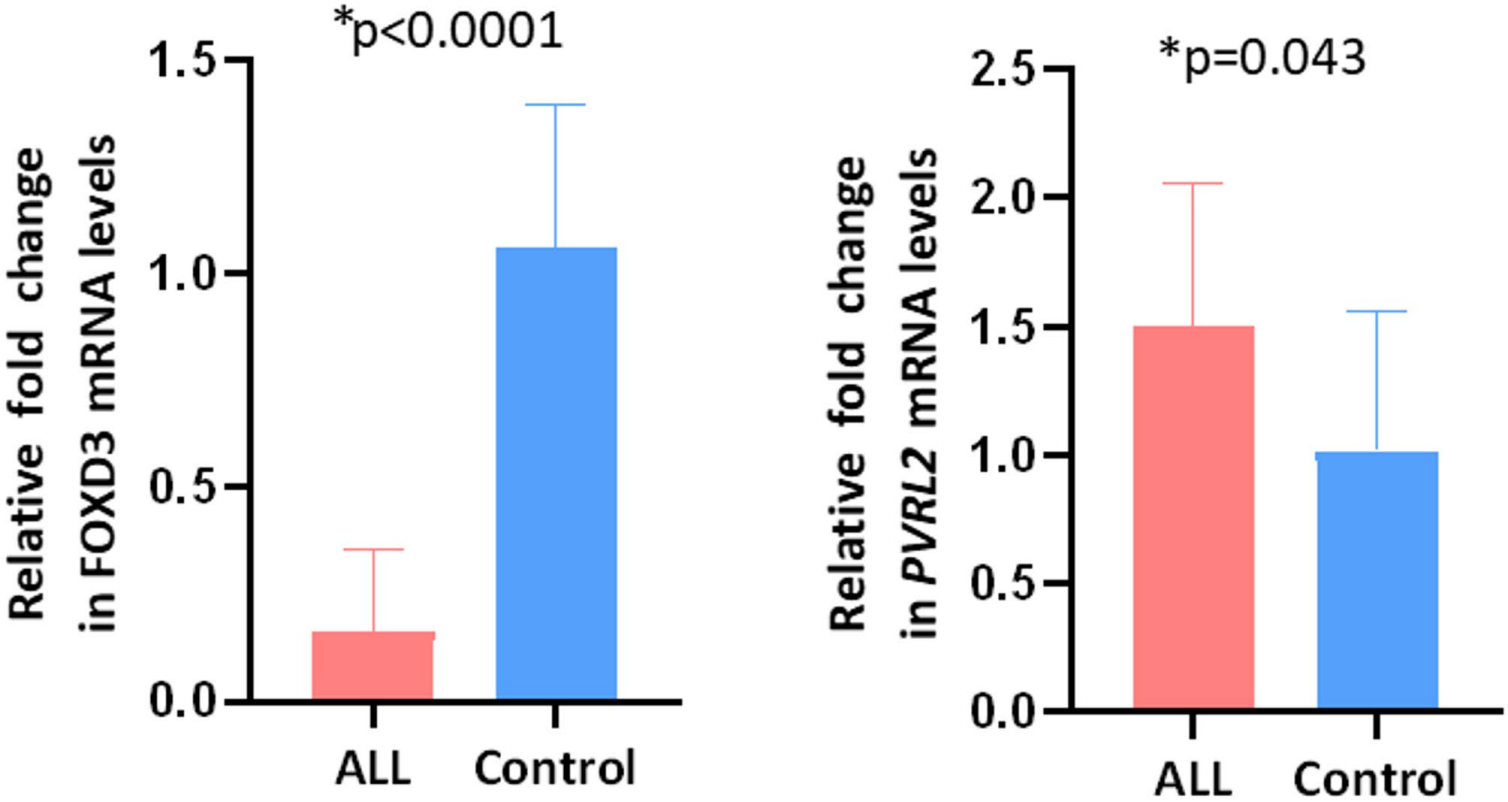
FOXD3 and PVRL2 mRNA expression in B-ALL patients and healthy controls. Bar plots showing the relative fold-change in mRNA expression of FOXD3 (left) and PVRL2 (right) in B-ALL patients (red) compared to healthy controls (blue). The bars represent the mean ± standard deviation (SD). Statistical analysis was performed using the Mann-Whitney U test. Statistical significance is denoted with *p < 0.05.

FOXD3, a downstream transcription factor known to negatively regulate VISTA expression, was analyzed in B-ALL patients. The relative fold change in FOXD3 mRNA expression was significantly lower in B-ALL patients compared to controls (0.1626 vs. 1.06; *p* < 0.0001), consistent with the observed overexpression of VISTA (Fig. 4).

In addition, the expression of PVRL2, an immune regulatory molecule, was analyzed in B-ALL patients using real-time PCR. The relative fold change in PVRL2 mRNA expression was slightly higher in B-ALL patients compared to controls (1.5 ± 0.54 vs. 1.02 ± 0.44, *p* = 0.043), as shown in Fig. 4, suggesting a possible role of PVRL2 in suppressing immune responses.

## Discussion

B-cell acute lymphoblastic leukemia (B-ALL) is characterized by significant immune dysregulation, which facilitates tumor survival and progression. In this study, we investigated the expression of VISTA, a novel immune checkpoint, and its interplay with other key immune regulatory markers, including CD244, CD48, FOXD3, and PVRL2, in pediatric B-ALL patients. Our findings reveal a complex immune suppressive network driven by the overexpression of VISTA and dysregulation of associated markers, highlighting their potential roles in immune evasion. These results advance our understanding of the immune microenvironment in B-ALL.

VISTA and CD244 are ICs that are overexpressed in many types of cancer^18,36^. They act as checkpoints for activated T cells, thus hindering their effector functions^37^. We analyzed VISTA on T helper, T cytotoxic, and blast cells in B-ALL.

Typically, VISTA is highly expressed on hematopoietic cells and leukocytes, especially the myelocytic lineage; lower expression on lymphoid lineage; T helper and T regulatory cells, to a lesser extent on T cytotoxic and NK cells^9^. It demonstrated an inhibitory effect on T cell proliferation and functions^9–12^. It inhibited the production of IL-2 by CD4^+^ and INF-γ by CD8^+ 11^. When being blocked, dendritic cells become activated, and a rise in IL-2 and TNF-α levels was observed^38^. Furthermore, blocking of VISTA by monoclonal antibodies reversed the exhaustion of cytotoxic T cells, INF-γ^+^CD8^+^ cells TILs increased, and CD8^+^ cells functions were restored^39^.

In AML, VISTA was found to be highly expressed on MDSCs. When knocked out, an improvement in CD8^+^ T cell proliferation was observed, suggesting an inhibitory role of VISTA on CD8^+^ T cell^40^. Moreover, targeting VISTA reversed VISTA-mediated immune suppression, and posed an anti-tumoral effect^41^. In AML, an increase in blasts expressing VISTA was reported^40^. In our study, VISTA was overexpressed on blasts when compared to controls. Also, its expression on blasts was higher within the NCR group than the CR group, suggesting a possible contribution of VISTA expression to the prognostic state of patients. Moreover, VISTA displayed a significantly increased expression on T helper and T cytotoxic cells. Moreover, VISTA acts as a coinhibitory receptor for CD4^+^ cells, contributes to tolerance, and prevents autoimmunity^42,43^.

It has been shown that VISTA is essential for the quiescence state of naïve T cells^44^. It is suggested that VISTA could drive the transition of naïve T cells to FOXP3^+^ T regs^9^. The FOXP3^+^ Tregs are increased in B-ALL patients and contribute to their immunosuppression^45–48^. Also, T regs become active and higher in frequency, thus further downregulating the activity of lymphocytes in AML and CLL^24,49–52^suggesting that the observed increase could be owed to an increase in T regs frequency however, further investigation is required. The increased VISTA expression on CD8^+^ cells observed in our work is consistent with previous findings which reported that VISTA is overexpressed on CD4^+^ and CD8^+^ cells. This increased VISTA expression could drive T cells to a state of dysfunction and diminished proliferation, as indicated by our work’s marked decrease in the CD3 population.

The transcription factor FOXD3 negatively regulates the expression of VISTA via the BRAF/MEK/ERK pathway in melanoma^22^. The silencing of FOXD3 was detected in the early phase B cell transition, followed by DNA methylation, which implies a key role in the process of leukemogenesis in CLL in both mouse models and humans^26^. Also, its mRNA level was found to be reduced in CLL^23^. A decreased expression of FOXD3 was also associated with poor prognosis in high-grade gliomas^25^and its silencing increased cancer cell proliferation in colon cancer^27^. It inhibited cancer cell proliferation and enhanced apoptosis in ovarian cancer, and its expression was reduced and hypermethylated^53^. Besides, high expression levels of VISTA in patients with glioma were associated with poor prognosis^54^. In line with these findings, FOXD3 expression was significantly lower in ALL in our study. Thus, we suggest a possible contribution of FOXD3 to the increased levels of VISTA-expressing cells in ALL.

Immune regulatory molecule CD244 is expressed by T cells and NK cells^28^. On NK cells it could be either stimulatory or inhibitory depending on several aspects including the frequency of surface CD244 expression, the extent of cross-linking with its ligand CD48, and the signaling pathway utilized^55^. Previous findings in AML reported a greater frequency of exhausted cytotoxic T lymphocytes expressing CD244 in bone marrow samples than in PB samples^36^. Increased expression of CD244 on T helper and T cytotoxic cells in AML and CLL was observed^30,56^. Despite these findings, our data reported no alteration in the expression of CD244. The discrepancy in surface expression of the CD244 can be attributed to the fact that, unlike the before mentioned studies, our samples were taken from the pediatric population, in addition to pathological and etiological differences between these diseases that may alter the phenotypic features of T cells.

CD48 is present in all hematopoietic cells, including T lymphocytes, B lymphocytes, and NK cells^32,57,58^. It is the ligand for CD244 ^31^. It is involved in the activation and regulation of immune cells^59^. It was downregulated in AML cells^60^. The interaction between CD244 and CD48 together with the extent of CD244 expression, shows a positive immunoregulatory effect on NK cells and plays a role in their activation and lysis^55^. Previous work detected reduced expression of CD48 by blasts in T-ALL^61^ and AML^60^. Similarly, in the current study, CD48 is downregulated. Based on this, the downregulation of CD48 by blast cells can aid their escape from NK-mediated cytotoxicity and subsequent elimination. This also was observed with CD48 expressing non-small cell lung cancer, which was more susceptible to NK-killing^62^. In agreement with this, many viral infections appear to adopt this mechanism, such as cytomegalovirus which showed a reduced CD48 expression by macrophages thus hindering itself from NK cells^63^.

Additionally, blast cells lose phenotypic features like CD48 expression, which is normally expressed by B cells and is involved in its activation^64^. This implies a possible reduction in B cell activation. In CLL, CD48 was downregulated by blasts with 11q chromosomal aberration when compared with blasts without this aberration^65^. This highlights the effect of different chromosomal abnormalities on the phenotypic features of leukemia cells.

Our data described an increased frequency of CD48^+^CD3^+^CD8^+^ cells, indicating a possible tendency toward CD8^+^ cell activation. However, ICs such as VISTA and TIGIT might eventually block this activation. Suggesting that CD48 could be upregulated by cytotoxic T lymphocytes as it normally becomes upregulated on subsets of activated T cells/memory CD8^+^ cells to promote TCR signaling and T cell activation^59,66,67^.

Our study highlights the complex immune regulatory network involved in B-cell acute lymphoblastic leukemia (B-ALL), focusing on the interplay between VISTA, CD244, CD48, FOXD3, and PVRL2. The overexpression of VISTA, particularly on CD8^+^ T cells and CD19^+^ B cells, suggests its pivotal role in immune suppression. VISTA is known to attenuate T-cell activation and promote exhaustion, which may contribute to the inability of cytotoxic T cells to mount effective anti-leukemic responses. This is further supported by the significant downregulation of FOXD3, a transcription factor that regulates VISTA expression. The reduced FOXD3 levels observed in our study may directly contribute to the observed overexpression of VISTA, highlighting a regulatory mechanism that warrants further exploration^18,36^.

The altered expression of CD48, the ligand for CD244, provides additional insights into the immune evasion strategies employed by leukemic cells. In B-ALL patients, CD48 was significantly downregulated on CD19^+^ B cells, potentially disrupting CD244-CD48 interactions. This disruption could impair the activation and cytotoxic functions of NK cells and CD8^+^ T cells, which rely on this interaction for efficient tumor recognition and elimination. In contrast, the upregulation of CD48 on CD8^+^ T cells may reflect a compensatory immune response to overcome suppression, although its functional impact in the context of an exhausted immune environment remains unclear^31^.

Further, the upregulation of PVRL2, an immune regulatory molecule, suggests its role in enhancing the suppressive tumor microenvironment. Elevated PVRL2 levels have been associated with interactions that inhibit immune cell activation, aligning with the immune dysfunction observed in B-ALL. Collectively, the interplay between PVRL2 upregulation, VISTA overexpression, and CD48 downregulation underscores a coordinated mechanism of immune suppression and evasion, allowing leukemic cells to thrive despite an activated but ineffective immune system^62^.

These findings underscore the potential of VISTA as a therapeutic target in B-ALL. The observed alterations in CD244-CD48 interactions and the regulatory influence of FOXD3 and PVRL2 further highlight the importance of a multi-targeted approach to restoring immune function in these patients. Future therapeutic strategies could focus on inhibiting VISTA, enhancing CD244-CD48 interactions, and modulating the expression of transcriptional and immune regulatory molecules such as FOXD3 and PVRL2 to reverse immune suppression and improve patient outcomes.

## Conclusion

Our study highlights the role of VISTA as an important immune checkpoint in B-cell acute lymphoblastic leukemia (B-ALL), demonstrating its overexpression on CD8 + T cells and CD19 + B cells, which contributes to immune suppression and tumor evasion. Moreover, VISTA was upregulated on blasts and cytotoxic T cells in the NCR group of patients, implying a possible contribution to the unfavorable prognostic state. The downregulation of FOXD3, along with the dysregulation of CD48 and PVRL2, underscores the complex immune evasion mechanisms in B-ALL. These findings suggest that targeting VISTA and its regulatory pathways, along with modulating CD244-CD48 interactions, may offer promising therapeutic strategies for B-ALL.

This study is limited by a small sample size and the absence of functional assays to validate the biological effects of marker dysregulation. Additionally, an extended analysis of these markers on other immune cells, such as monocytes and NK cells, could provide valuable insights. Future research should address these limitations by incorporating larger cohorts, performing functional studies, and including adult patient populations to further validate and generalize these findings.

## Electronic supplementary material

Below is the link to the electronic supplementary material.

Supplementary Material 1

## Data Availability

Datasets generated during the current study are available on request from the corresponding author.
