# Supplementary material for: VISTA is a potential target for immunotherapy in B-cell acute lymphoblastic leukemia in children
Source: Sci Rep. 2025 Jul 3;15:23809. doi: 10.1038/s41598-025-08164-2 (PMC12229706; doi:10.1038/s41598-025-08164-2)
Supplement: Supplementary file 1 — Supplementary Material 1 [file 41598_2025_8164_MOESM1_ESM.docx]

**Title**

**VISTA is a potential target for immunotherapy in B-cell acute lymphoblastic leukemia in children**

Nourhan K Mohamed^1,2^, Mohamed A El-Mokhtar^3^, Asmaa M Zahran^4^, Gamal. F M Gad^1^, Reham Ali Ibrahem^1^

**
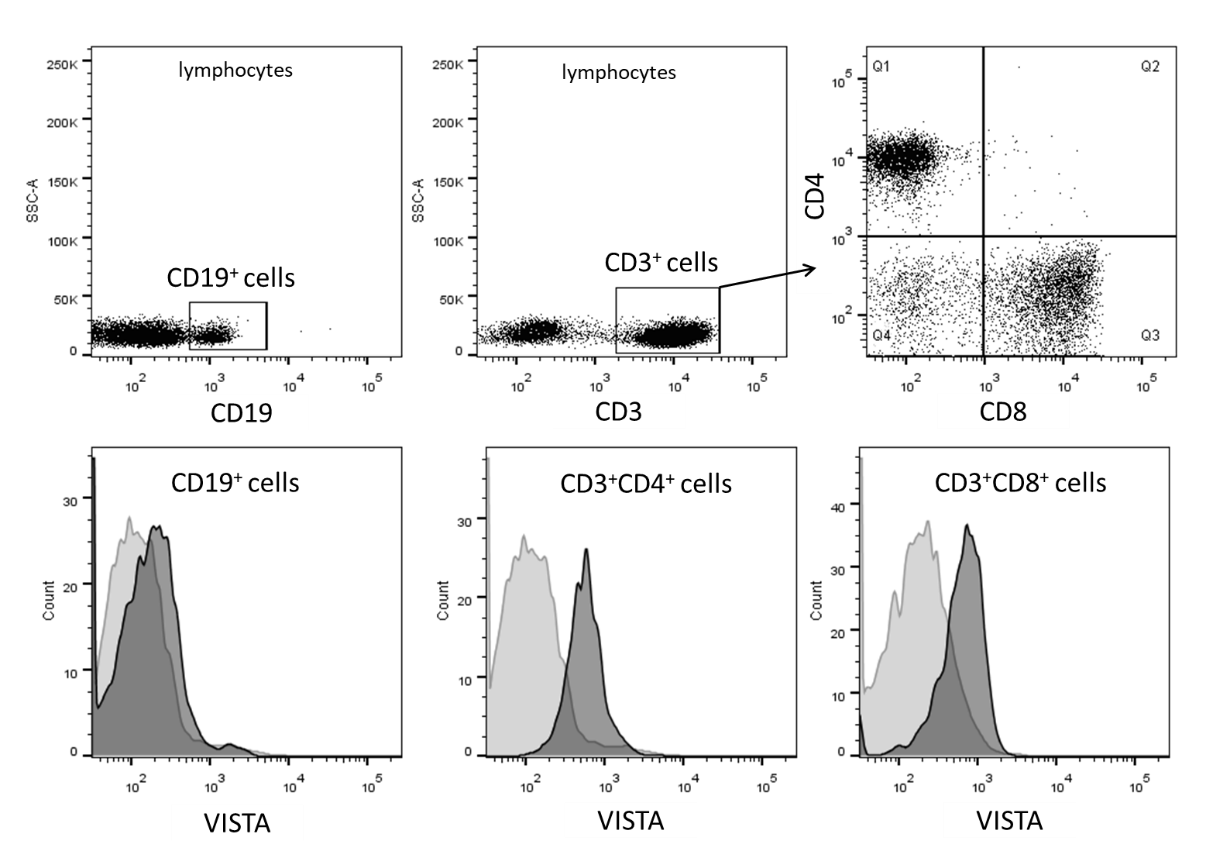
**

**Supplementary Figure 1. Gating strategy for the main lymphocyte subsets and VISTA expression.** Representative flow cytometry plots demonstrating the gating strategy used to identify lymphocyte subsets in B-ALL patients. (Top row) Gating for CD19+ B cells, CD3+ T cells, and further sub-gating of CD3+ cells into CD3+CD4+ T helper cells (Q1), CD3+CD8+ cytotoxic T cells, and double-negative (CD3+CD4-CD8-) or double-positive (CD3+CD4+CD8+) subsets. (Bottom row) Histograms show the expression of VISTA on CD19+ B cells, CD3+CD4+ T cells, and CD3+CD8+ T cells. Gray histograms represent isotype controls, and black histograms represent VISTA expression.
